# Epidemiology of Microbial Keratitis in Uganda: A Cohort Study

**DOI:** 10.1080/09286586.2019.1700533

**Published:** 2019-12-12

**Authors:** Simon Arunga, Guyguy M. Kintoki, James Mwesigye, Bosco Ayebazibwe, John Onyango, Joel Bazira, Rob Newton, Stephen Gichuhi, Astrid Leck, David Macleod, Victor H. Hu, Matthew J. Burton

**Affiliations:** a International Centre for Eye Health, London School of Hygiene & Tropical Medicine, London, UK; b Department of Ophthalmology, Mbarara University of Science and Technology, Mbarara, Uganda; c Department of Microbiology, Mbarara University of Science and Technology, Mbarara, Uganda; d Ruharo Eye Centre, Ruharo Mission Hospital, Mbarara, Uganda; e Department of epidemiology, Uganda Virus Research Institute, Entebbe, Uganda; f Department of Ophthalmology, University of Nairobi, Nairobi, Kenya; g Tropical Epidemiology Group, London School of Hygiene & Tropical Medicine, London, UK

**Keywords:** Microbial keratitis, bacterial keratitis, fungal keratitis, keratitis, blindness, Uganda

## Abstract

**Purpose:**

To describe the epidemiology of Microbial Keratitis (MK) in Uganda.

**Methods:**

We prospectively recruited patients presenting with MK at two main eye units in Southern Uganda between December 2016 and March 2018. We collected information on clinical history and presentation, microbiology and 3-month outcomes. Poor vision was defined as vision < 6/60).

**Results:**

313 individuals were enrolled. Median age was 47 years (range 18–96) and 174 (56%) were male. Median presentation time was 17 days from onset (IQR 8–32). Trauma was reported by 29% and use of Traditional Eye Medicine by 60%. Majority presented with severe infections (median infiltrate size 5.2 mm); 47% were blind in the affected eye (vision < 3/60). Microbiology was available from 270 cases: 62% were fungal, 7% mixed (bacterial and fungal), 7% bacterial and 24% no organism detected. At 3 months, 30% of the participants were blind in the affected eye, while 9% had lost their eye from the infection. Delayed presentation (overall *p* = .007) and prior use of Traditional Eye Medicine (aOR 1.58 [95% CI 1.04–2.42], *p* = .033) were responsible for poor presentation. Predictors of poor vision at 3 months were: baseline vision (aOR 2.98 [95%CI 2.12–4.19], *p* < .0001), infiltrate size (aOR 1.19 [95%CI 1.03–1.36], *p* < .020) and perforation at presentation (aOR 9.93 [95% CI 3.70–26.6], *p* < .0001).

**Conclusion:**

The most important outcome predictor was the state of the eye at presentation, facilitated by prior use of Traditional Eye Medicine and delayed presentation. In order to improve outcomes, we need effective early interventions.

## Background

Microbial keratitis (MK) can be caused by a range of pathogens including, bacteria, viruses, protozoa, and fungi. It is characterized by acute or sub-acute onset of pain, conjunctival hyperemia, and corneal ulceration with a stromal inflammatory cell infiltrate.^1^

MK has been described as a “silent epidemic”, which leads to substantial morbidity, related to blindness, pain, and stigma.^2^ It is the leading cause of unilateral blindness after cataract in Tropical regions estimated at 2 million cases of monocular blindness per year.^3^ In 2017, 1.3 million individuals were bilaterally blind from corneal opacity globally (excluding trachoma and vitamin A deficiency), accounting for 3.2% of the binocular blindness.^4^ In Sub-Saharan Africa (SSA), MK is an important cause of binocular blindness and is responsible for about 15% of the monocular blindness (Nigeria National Survey).^5,6^ The only report of the incidence in SSA is from Malawi in 1994, which suggested a rate of around 180/100,000/year.^7^ Rates in high-income settings are lower at 5–10/100,000.^8–10^

MK frequently leads to sight-loss from dense corneal scarring, or even loss of the eye, especially when the infection is severe and/or appropriate treatment is delayed. A good outcome depends on early appropriate treatment, supported by correct identification of the causative organism, and careful follow-up.^11,12^ In low and middle-income countries (LMIC), these resources are not readily available and outcomes tend to be poor.^13^

Literature on MK in SSA is extremely sparse, only one audit from an LMIC setting (Tanzania) has previously reported outcomes of MK at discharge in SSA.^13^ Here, in this large prospective cohort study from South-Western Uganda, we describe patient presentation, causative organisms, 3-month outcomes, and investigate their determinants.

## Methods

### Ethical statement

This study followed the tenets of the Declaration of Helsinki. It was approved by the London School of Hygiene & Tropical Medicine Ethics Committee (Ref 10647), Mbarara University Research Ethics Committee (Ref 10/04-16) and Uganda National Council for Science and Technology (Ref HS-2303). Written informed consent in the local language was obtained before enrolment. If the patient was unable to read, the information was read to them, and they were asked to indicate their consent by application of their thumbprint, which was independently witnessed.

### Study design and setting

In this cohort, we prospectively enrolled patients with MK that presented to Ruharo Eye Centre (REC) and Mbarara University and Referral Hospital Eye Centre (MURHEC) from December 2016 to March 2018. MURHEC is a government-owned tertiary eye unit established in 2013. It provides mostly free services and sees about 6,000–10,000 patients/year. REC is a church-run fee-paying tertiary eye hospital founded in the 1960s. It sees about 20,000–25,000 patients/year. Both hospitals are located in Mbarara Municipality, South-Western Region, Uganda. In order to investigate the seasonal variation in the presentation of MK, we aimed to recruit all MK cases presenting during at least one year.^13^

### Study participants

MK was defined as loss of corneal epithelium (of at least 1-mm diameter) with underlying stromal infiltrate, associated with any or all signs of inflammation (conjunctival hyperemia, anterior chamber inflammatory cells, ± hypopyon).^14^ We also included patients presenting with a deep corneal abscess (of at least 1 mm), defined as having all the features of MK, but without an epithelial defect. We excluded those not willing to participate, those not willing to return for follow-up, pregnant women, lactating mothers and those aged below 18 years.

### Assessment

We documented basic demographic information and their ophthalmic history. This included the circumstances in which their eye became infected, predisposing factors, treatment received, and their “health care journey” before reaching the eye hospital. Presenting Log MAR (Logarithm of Minimum Angle of Resolution) visual acuity at 2 m in a dark room was measured using Peek Acuity software.^15^ Participants were examined with a slit lamp to assess the anterior segment using a structured protocol, including eyelid assessment, corneal ulcer features, anterior chamber (flare, cells, hypopyon shape, and size) and perforation status. Infiltrate size was determined from the greatest diameter of the infiltrate (major axis) and the widest perpendicular diameter (minor axis).^14^ The final infiltrate size was then derived as the geometric mean of these two diameters.^14^ The same was repeated after fluorescein staining of the ulcer to determine epithelial defect sizes. High-resolution digital photographs with and without fluorescein staining were taken with a Nikon SLR 7200 digital camera with Macro lens.

Corneal scrape specimens were collected from the ulcer at a slit lamp or an operating microscope, using 21G needles after application of a proxymetacaine (minims) anesthetic eye drops. Samples underwent processing for the Gram stain, Potassium Hydroxide [KOH] stain, Calcofluor White [CFW] stain and direct inoculation on culture media (Sheep’s Blood Agar [BA], Chocolate Agar [HBA], Potato Dextrose Agar [PDA] and Brain Heart Infusion broth [BHI]). Two sterile corneal swab samples were taken for pan fungal gene sequencing. The number of corneal samples was dependent on how much material could be safely scraped from the cornea. The order was samples for microscopy, agar, broth, and finally corneal swabs.

In addition, a random blood sugar test and HIV counseling and testing were offered, as per the Uganda Ministry of Health HIV testing protocol. For those who were confirmed as HIV positive, a CD4 test was performed to determine the level of immune suppression and they were referred to the HIV care center, which is on the hospital site.

Microscopy, culture, and antimicrobial sensitivity work were done at the Mbarara University Department of Microbiology. The technician underwent initial training in ocular microbiology at the Aravind Eye Hospital System, department of ocular Microbiology in Madurai, India and had a site supervision visit by a mycologist from the London School of Hygiene & Tropical Medicine. Immediate CFW staining was also done in the side lab at MURHEC on a fluorescein microscope (Zeiss Primostar ILED) by the attending ophthalmologist. Agar plates and broths were incubated and read daily at 35–37°C for bacteria for up to 7 days and at 25°C for up to 21 days for fungi. Organism identification and sensitivity testing (MIC/zone of inhibition) were performed using standard microbiological techniques. We followed a previously described approach for reporting positive microbiology results.^16^ Briefly, bacteria were identified using routine biochemical identification tests. Identification of fungi was according to the macroscopic appearance of cultures on potato dextrose and microscopic appearance of conidia and spore-bearing structures. Positive culture was growth at the site of inoculation or growth on one solid medium consistent with microscopy; or semiconfluent growth at the site of inoculation on one solid medium (if bacteria); or growth of the same organism on repeated scraping. If, by microscopy, hyphae were observed in corneal tissue, but failed to grow in culture, the causative organism was reported as fungal.

### Treatment and follow-up

Patients were treated empirically at presentation and the treatment choice was reviewed when the microbiology results became available. Patients with fungal keratitis were treated with Natamycin 5% eyedrops (Zonat Sunways India), those with bacterial keratitis were treated with Ofloxacin 0.3% eyedrops (Biomedica Remedies-India). Patients with fungal infection were treated hourly day and night for the first 3 days and then hourly while the patient was awake for 2 weeks. This was changed to 2-hourly for another 2 weeks and then tapered to 4 times a day until healed. For bacterial infections, patients were treated hourly day and night for the first 3 days and then reduced to 6 times a day for a further week. All patients with fungal MK were also given Ofloxacin 0.3% eye-drops four times a day as prophylaxis until all epithelial defects were healed. In addition, those in pain were treated with Atropine 1% eye-drops (locally formulated) and oral Paracetamol tablets. Raised intraocular pressure was treated with Timolol 0.5% eye-drops (locally formulated). Those with presumed viral keratitis were treated with Acyclovir 3% eye ointment (CIPLA India) five times a day for 3 weeks. Most patients were admitted during the first week.

After the initial assessment patients were seen on day 2, day 7, day 21, and day 90 (3 months). Additional assessments were conducted as clinically indicated. The main outcome measures were final best-corrected vision at 3 months, blindness (<3/60 in the affected eye) at 3 months, and loss of the eye at 3 months. Scar density was also graded as “no scar” (clear cornea), “mild scar” (anterior chamber structures clearly visible through the scar), “moderate scar” (anterior chamber structures vaguely visible through the scar) and “dense scar” (anterior chamber structures completely obscured by the scar).

### Analysis

Data were analyzed in STATA v14. To describe the presentation of MK, summary frequency tables of demographics, presentation time, clinical history and clinical features were generated. Presentation time was classified as prompt (0–3 days), early (4–7 days), intermediate (8–14 days), late (15–30 days) and very late (more than 30 days).^17^ In addition, a summary tally of patients that presented by month across one year (2017) was generated to describe the presentation pattern. This was compared to local rainfall, humidity and temperature patterns. Local weather data were obtained from the weather and climate repository.^18^ For presentation purposes, Log MAR visual acuity measurements were converted to the Snellen scale and categorized according to the WHO classification system.^19^

We used two different analytical approaches. We first took a causal modeling approach to explore the association of six risk factors of interest with visual acuity at presentation. These six factors were (Traditional Eye Medicine) TEM use, history of trauma, delayed presentation, distance from hospital, distance from nearest health center (HC), and organism type. In order to inform our modeling choices, we first drew Direct Acyclic Graphs (DAGs), using www.daggity.net v2.3 software, to identify relevant variables to adjust for in the multivariable logistic regression model.^20^ A DAG is a representation of the hypothesized order of events from the exposure to the outcome. It allows the researcher to logically map out relationships between different variables and identify those to adjust for to determine the overall effect of the exposure on the outcome. A change in point estimate criteria was used to assess for confounding and multi-collinearity. Each main exposure was separately adjusted for confounding factors and final adjusted odds ratios (aOR) recorded.

The second modeling approach was to build a predictive model for visual acuity outcomes at 3 months, using baseline clinical features. Patients without 3-month data were excluded from the analysis. Ordinal logistic regression analysis of the WHO Snellen visual acuity categories was used to identify factors associated with visual acuity at 3 months. Univariable regression was performed to generate crude odds ratios (cOR). Variables with a *p*-value less than 0.1 were initially included in the multivariable model. A backward stepwise approach was then used until only the variables with a *p*-value of less than 0.05 were retained. Adjusted ORs were reported for the final model.

## Results

### Participants

**Figure 1.**
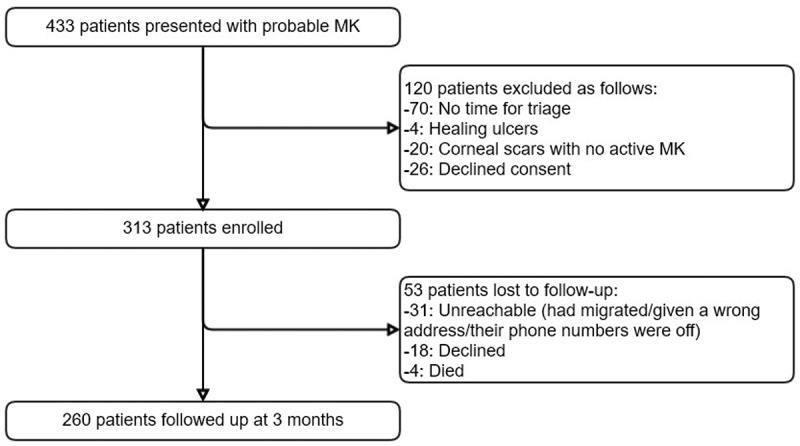
Flow diagram of participants who were enrolled in the cohort study.

Patient enrolment is illustrated in Figure 1. The baseline characteristics of the patients are shown in Table 1. Median age was 47 years (IQR 35–60, total range 18–96 years), and the majority (56%) were male. Over a quarter had never had any formal education. Most (70%) were married and most (70%) were the heads of households. Median distance from home to the eye hospital was 79 km (IQR 52–128, total range 0.2–378 km). Median distance from home to their nearest HC was 3 km (IQR 1–4, total range 0–45 km). The main occupation was farming (70%). The baseline characteristics of the patients who were lost to follow-up and those who completed 3 months were similar (Supplementary Table 1).

**Table 1.** Demographic characteristics of the study participants.

Variable	n/313	(%)
**Age (median = 47, IQR 35–60) in years**		
< 30 years	54	(17%)
30–40 years	63	(20%)
40–50 years	59	(19%)
50–60 years	66	(21%)
> 60 years	71	(23%)
**Gender**		
Female	139	(44%)
Male	174	(56%)
**Occupation**		
Farmer	220	(70%)
Non-farmer	93	(30%)
**Education**		
None	84	(27%)
Primary level	162	(52%)
Secondary level	45	(14%)
Tertiary level	22	(7%)
**Marital status**		
Unmarried^a^	95	(30%)
Married	218	(70%)
**Economic status^b^**		
Lower	85	(28%)
Middle	189	(63%)
Upper	26	(9%)
**Being head of household**		
Yes	212	(68%)
No	101	(32%)
**Distance from the eye hospital (median = 79 km IQR 52–128)**		
0–50 km	77	(25%)
50–100 km	111	(35%)
100–150 km	75	(24%)
>150 km	50	(16%)
**Nearest health center (Median 3 km, IQR 1–4 km)^c^**		
Clinic	10	(3%)
HC II	103	(33%)
HC III	96	(31%)
HC IV	43	(14%)
Hospital	32	(10%)
Don’t know	29	(9%)

^a^Unmarried included single divorced and widowed.

^b^Economic status was self-reported where participants compared themselves with their neighborhood as “poor”, “neither poor nor rich” or “rich”, n was 300 with 13 non-reported values.

^c^The nearest health center was the health center that the patients considered nearest to them regardless of the level of that health center

### Presentation pattern

**Figure 2.**
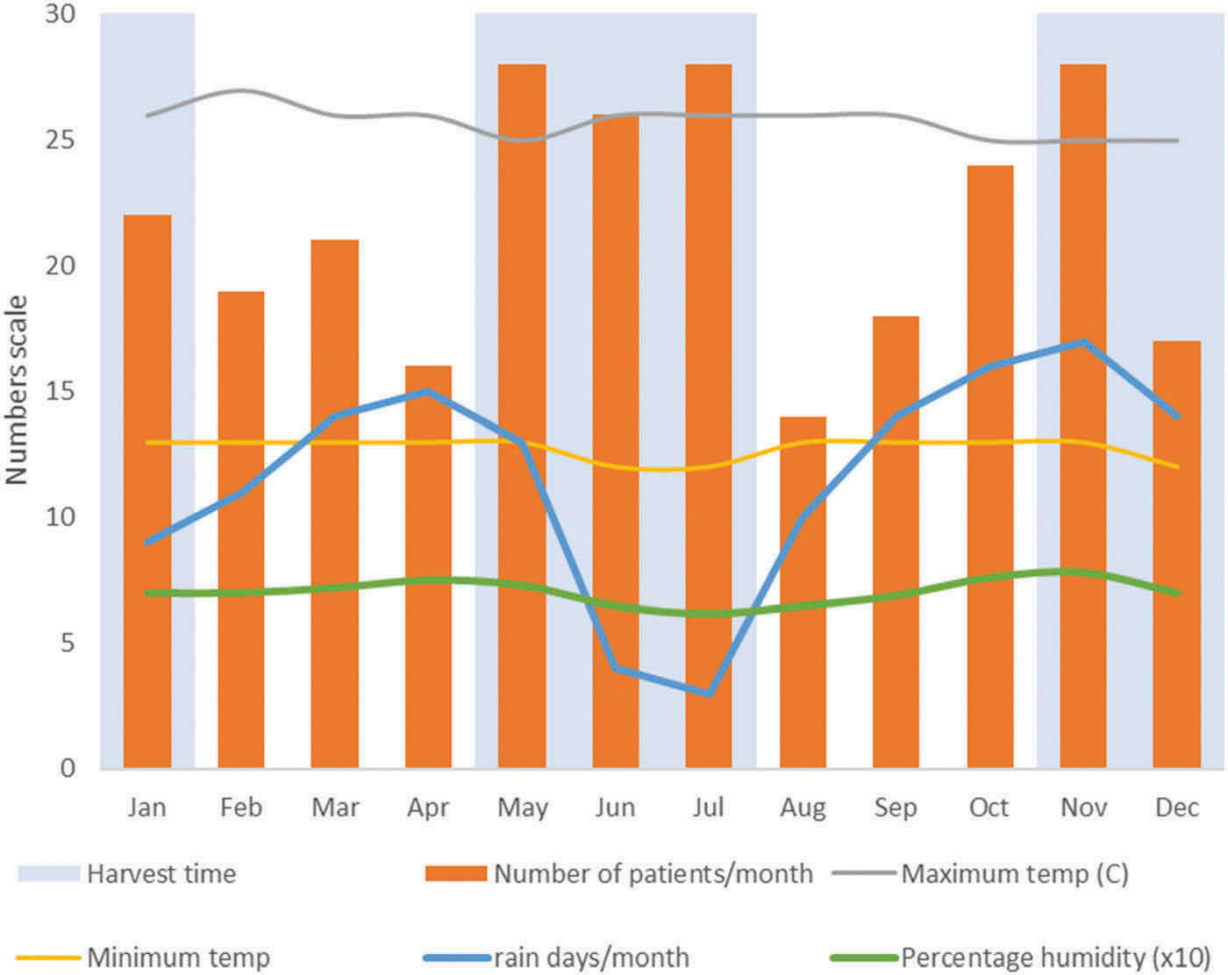
Presentation of patients with MK, by month in 2017 (n = 261). Monthly average minimum and maximum temperatures, average humidity and the number of days with rain are overlaid. Humidity was in percentage but was scaled to tens (divided by 10) to fit on the plot scale.

Figure 2 illustrates the number presenting per month throughout 2017, compared to rainfall, temperature and humidity patterns. Patients presented throughout the year, with peaks in May to July and October to November, which corresponded with the harvest seasons. April and November had the greatest rainfall. Temperature and humidity were constant throughout the year.

### Presenting history

The median time from onset of symptoms to presentation time at the eye unit was 17 days (IQR 8–32, total range 0–370 days), Table 2. Only 7% of the participants presented “promptly” (within 3 days). Only 29% of the participants reported a history of trauma, and most (74%) of these were classified as organic in nature. Many patients (60%) reported use of TEM.

**Table 2.** Clinical history.

Variable	n/313	(%)
**Presenting time (median = 17 days, IQR 8–32)^a^**		
Prompt 0–3 days	23	(7%)
Early 4–7 days	46	(15%)
Intermediate 8–14 days	72	(23%)
Late 15–30 days	79	(26%)
Very late >30 days	90	(29%)
**Most important symptom (self-reported)**		
Pain	144	(46%)
Reduced vision	137	(44%)
Other	32	(10%)
**History of trauma**		
Yes	91	(29%)
No	220	(71%)
**Used traditional eye medicine**		
Yes	188	(60%)
No	125	(40%)
**Used other treatment^b^**		
Yes	275	(88%)
No	38	(12%)
**Diabetic (n = 280)^c^**	22	(8%)
**HIV positive (n = 284)^c^**	37	(13%)

^a^n was 310. For 3 patients the date of onset could not be well ascertained.

Some patients had used other forms of eye drops prior to presentation and there was some overlap among those who used TEM and other eye drops.

^b^It was not possible to ascertain the forms of other treatment used.

^c^Some patients declined to be tested for HIV and diabetes

### Clinical features and microbiology

Table 3 shows the clinical features at presentation, including detailed characteristics of the ulcers and microbiology results. Specimen for microbiology was collected in 270 patients. Due to limited amounts of sample material, it was not possible to perform all tests on all those sampled. Almost half of the participants (47%) had a visual acuity of less than 3/60 (blind) in the affected eye at presentation. Microbiology results were available in 270/313 (86.3%) participants. Corneal scrapping was not performed on 43 participants who either did not consent, had deep-seated infiltrates, or small infiltrates (less than 0.5 mm). Overall, most infections were fungal (62%), 7% were bacterial and 7% were mixed (fungal and bacterial). Fifty-seven (20%) of the corneal scrapping samples were negative on both microscopy and culture.

**Table 3.** Clinical features and diagnosis at presentation (n = 313).

Variable	Median	(IQR [Total Range])
**Infiltrate size (mm)^a^**	5.2	(3.3–7.7 [0.5–13])
**Epithelial defect size (mm)^a^**	3.9	(2.4–6.5 [0–14])
Variable	n/313	(%)
**Snellen Visual Acuity in affected eye(n = 312)**		
6/5–6/18	102	(33%)
6/24–6/60	42	(12%)
5/60–3/60	24	(8%)
2/60–1/60	33	(11%)
Counting fingers-light perception	103	(33%)
No light perception	9	(3%)
**Snellen visual acuity in non-affected eye (n = 312)**		
6/5–6/18	278	(89%)
6/24–6/60	16	(5%)
5/60–3/60	2	(1%)
2/60–1/60	4	(1.2%)
Counting fingers-light perception	6	(2%)
No light perception	6	(1.8%)
**Slough (n = 312)^b^**		
No slough	62	(20%)
Flat	124	(40%)
Raised	126	(40%)
**Infiltrate edge (n = 293)**		
Defined	35	(12%)
Serrated	258	(82%)
Not visible	20	(6%)
**Satellite lesions present (n = 304)**		
Yes	178	(57%)
No	126	(40%)
**Infiltrate colour (n = 288)**		
White	148	(47%)
Cream	106	(34%)
Other colour	34	(11%)
**Hypopyon (median height 1.3mm IQR 0.9–2.9, n = 301)**		
Yes	94	(30%)
No	217	(69%)
**Site of ulcer (n = 310)^c^**		
Peripheral	27	(9%)
Paracentral	64	(21%)
Central	219	(70%)
**Perforation status**		
Not perforated	237	(76%)
Impending	31	(10%)
Perforated	48	(12%)
Perforated & sealed	7	(2%)
**Overall Laboratory diagnosis (n = 270)^d^**		
Unknown	65	(21%)
Bacterial	20	(6%)
Fungal	168	(54%)
Mixed (bacteria/fungal)	17	(5%)

Where n < 313 was due to some missing data: percentages calculated for 313 and rounded off to the nearest whole number.

**^a^**These were calculated as the geometrical means using the MUTT protocol. The upper limits exceeded normal corneal diameter for some lesions, which extended up to the sclera.

^b^Raised slough was when the corneal infiltrate profile was raised, flat slough was when the profile was flat while no slough is when there was no debris noted.

^c^Site of ulcer was peripheral when the ulcer was marginal, paracentral was when the ulcer was not marginal but not within 4 mm of the center of the cornea, central was when the ulcer was within the central 4 mm of the cornea.

Impending perforation is when the clinicians felt the ulcer would perforate in the next 48 h.

^d^Specimen for microbiology was collected in 270 patients. Due to limited amounts of sample material, it was not possible to perform all tests on all those sampled. The order of material collection was 3 slide smears (gram, KOH, CFW), 3 agar inoculations (blood, chocolate, PDA) and 1 broth (BHI) depending on available material.

### Outcomes

Table 4 shows the outcomes of the 260 participants seen at the 3-month follow-up. At 3 months, the visual acuity was better than baseline vision. Median final visual acuity (Log MAR) was 0.4 (IQR 0–1.5) compared to a baseline median of 1.3 (IQR 0.3–2.5). Visual acuity at 3 months improved in 139 participants, worsened in 66 participants and remained unchanged in 56 (sign rank test *p* < .0001). Visual acuity was categorized according to the WHO classification system and poor outcome was considered as vision < 6/60.^19^ Thirty percent of the participants were blind in the affected eye (vision less than 3/60) and 9% had lost their eye to infection due to evisceration following endophthalmitis.

**Table 4.** Outcomes at 3 months.

Variable	n/260	(%)
**Visual acuity in the affected eye (Snellen)**		
6/5–6/18	138	(53%)
6/24–6/60	37	(14%)
5/60–3/60	7	(3%)
2/60–1/60	14	(5%)
Counting fingers-light perception	31	(12%)
No light perception	33	(13%)
**Visual acuity in the non-affected eye**		
6/5–6/18	229	(90%)
6/24–6/60	11	(4%)
5/60–3/60	2	(1%)
2/60–1/60	0	(0%)
Counting fingers-light perception	6	(2%)
No light perception	7	(3%)
**Outcome**		
Healed no scar	34	(12%)
Healed mild scar	83	(30%)
Healed moderate scar	65	(24%)
Healed dense scar	46	(17%)
Eviscerated	24	(9%)
Not healed	20	(7%)
Staphyloma	4	(1%)

### Causal modeling for poor presentation

**Figure 3.**
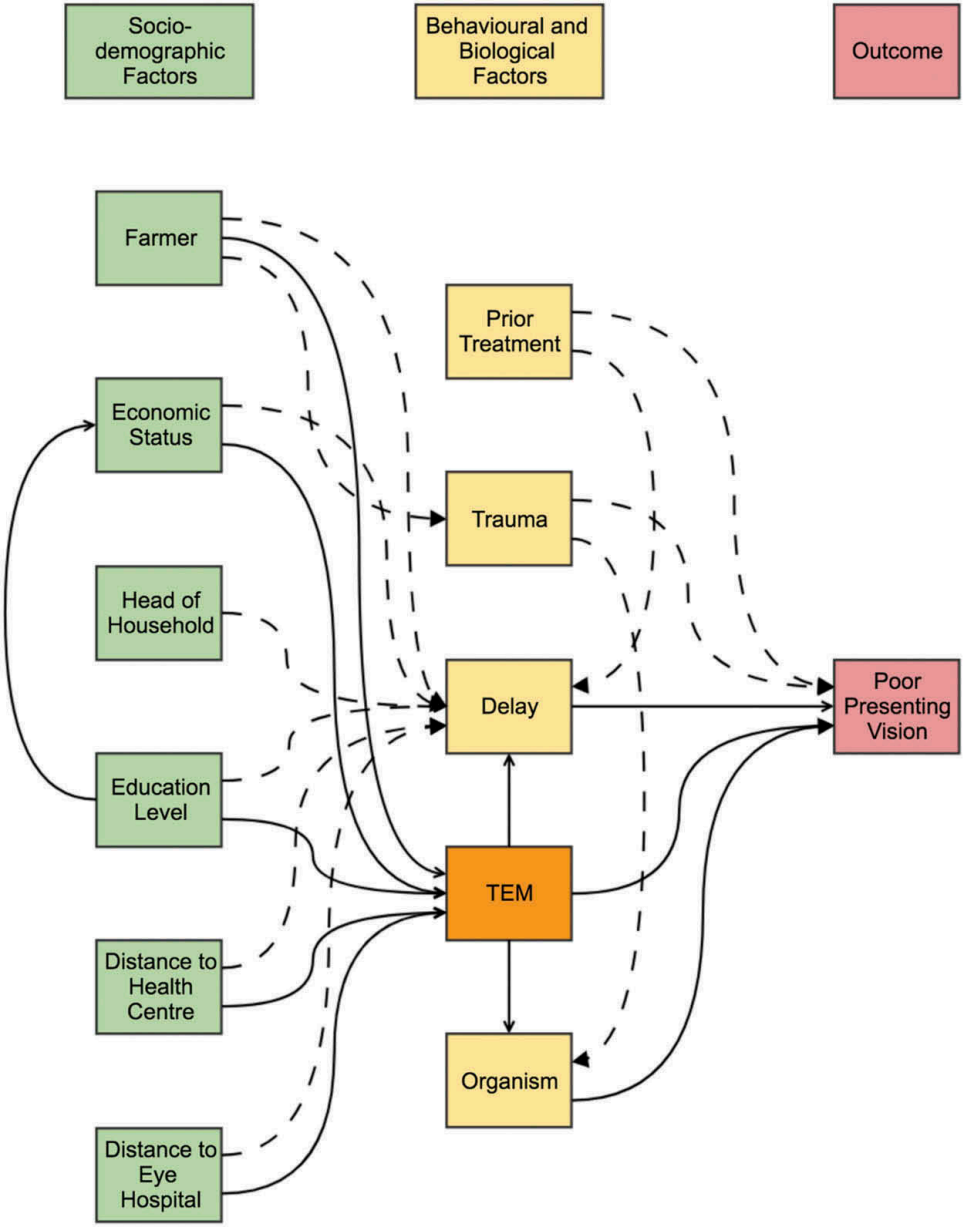
A DAG framework showing the causal pathways for poor presenting vision. This diagram is adjusted to illustrate the role of TEM. The solid lines indicate hypothesized direct relationships and the dashed lines indicate hypothesized indirect relationships.

Figure 3 shows the overall model for several variables of interest that we considered in the causal analysis for poor presenting vision. The results are summarized in Table 5 and their corresponding outputs from the DAGitty software in Supplementary Figures 1–5. Those who reported TEM were estimated to have overall 1.6 times the odds of being in a poorer vision category compared to those who did not use TEM (aOR 1.62 [95%CI 1.04–2.54], *p* = .033). It was considered plausible that some of this effect was mediated through delayed presentation and/or organism type, and after adjusting for these factors as well, the aOR was 1.47 [95%CI 0.91–2.38], *p* = .11. There was some evidence (*p* = .033) of an association between the category of presentation time and presenting vision, with the lowest odds of poorer vision being in those that present earliest and increasing odds as delay increases. No evidence (*p* = .609) was found of an association between trauma and presenting visual acuity, but strong evidence was found of an association between presenting visual acuity and both distance from the eye hospital (*p* < .001) and distance from the nearest HC (*p* = .007). Interestingly, even after adjusting for delayed presentation there remained strong evidence of an association (*p* < .0001 and *p* = .009).

**Table 5.** Causal modeling for poor presenting vision (n = 313).

Variable	Univariable Analysis			Multivariable Analysis			Multivariable analysis for direct effect		
	Crude OR^a^	(95% CI)	p-value	Adj. OR	(95% CI)	p-value	OR	(95% CI)	p-value
**Model 1: Used Traditional Eye Medicine (TEM) as the main exposure of interest^b^**									
Used traditional eye medicine (TEM)	1.78	(1.17–2.70)	0.007	1.62	(1.04–2.54)	0.033	1.47	0.91–2.38	0.11
**Model 2: Delayed presentation as the main exposure of interest^c^**									
Prompt 0–3 days	1		0.0004	1		0.033			
Early 4–7 days	2.78	(1.07–7.20)		1.94	(0.70–5.39)				
Intermediate 8–14 days	4.45	(1.84–10.7)		3.02	(1.17–7.79)				
Late 15–30 days	5.58	(2.33–13.3)		3.57	(1.40–9.07)				
Very late > 30 days	2.63	(1.11–6.25)		1.87	(0.74–4.72)				
**Model 3: Trauma as the main exposure of interest^d^**									
Positive history of trauma	0.94	(0.60–1.48)	0.810	1.13	(0.70–1.83)	0.609			
**Model 4: Distance from the eye hospital in km as the main exposure of interest^e^**									
0–50 km	1		< 0.0001	1		< 0.0001	1		< 0.0001
50–100 km	1.27	(0.73–2.21)		1.27	(0.73–2.21)		1.26	(0.71–2.21)	
100–150 km	2.90	(1.60–5.23)		2.90	(1.60–5.23)		2.63	(1.45–4.80)	
> 150 km	5.60	(2.87–10.9)		5.60	(2.87–10.9)		5.06	(2.58–9.92)	
**Model 5: Distance from nearest health center (for every km increase)^e^**									
Distance from nearest health center (for every km increase)	1.22	(1.05–1.41)	0.007	1.22	(1.05–1.41)	0.007	1.21	(1.05–1.41)	0.09
Model 6: Type of organism^f^									
No organism detected	1		0.105	1		0.101			
Bacteria	1.25	(0.50–3.15)		1.43	(0.55–3.66)				
Fungal	1.80	(1.05–3.07)		1.82	(1.06–3.13)				
Mixed	2.49	(0.93–6.62)		2.71	(1.07–2.79)				

^a^All crude estimates were adjusted for age and sex. ^b^Use of TEM was adjusted for age, sex, being a farmer, economic status, education level, distance from the eye hospital, and distance from the nearest health center (n = 298). After adjusting for delay and organism type, the effect of TEM was OR 1.47 95% CI 0.91–2.38, *p* = 0.11. ^c^Delayed presentation was adjusted for age, sex, being a farmer, distance, economic status, education level, TEM, trauma, and previous use of prior treatment before presentation (n = 295). ^d^History of trauma was a priori based on literature from previous studies. It was adjusted for age, sex, being a farmer, TEM, distance, and prior treatment (n = 306). ^e^Long distance from the eye hospital and long distance from the nearest health center were only adjusted for age and sex (n = 309). Their crude and adjusted point estimates are the same. However, the direct effect of distance to eye hospital and distance to nearest health center after adjusting for delay was still highly significant, *p* < 0.0001 and = 0.009. ^f^Type of organism was a forced priori and was adjusted for trauma and use of TEM (n = 267).

### Predictors of outcome

In the final multivariable model, worse visual acuity outcome at 3 months was associated with baseline vision, size of the infiltrate and perforation status at presentation Table 6.

**Table 6.** Factors at presentation predictive of a poor final visual acuity (WHO snellen ordinal scale) at 3 months (n = 260).

Variable	Univariate Analysis			Multivariable Analysis		
	Crude OR^a^	(95% CI)	p-value	Adjusted OR^b^	(95% CI)	p-value
**Baseline visual acuity** (for every line decrease in vision)	4.78	(3.59–6.35)	< 0.0001	2.98	(2.12–4.19)	< 0.0001
**Presence of slough**						
None	1		0.007			
Flat	1.91	(0.95–3.83)				
Raised	2.95	(1.46–5.95)				
**Infiltrate edge being serrated**	0.84	(0.58–1.24)	0.393			
**Satellite lesions being present**	0.64	(0.40–1.03)	0.068	0.51	(0.28–0.90)	0.021
**Infiltrate color**						
White	1		< 0.0001			
Cream	2.70	(1.56–4.63)				
Colored	6.37	(3.10–13.2)				
**Hypopyon present**	2.16	(1.38–3.55)	0.002			
**Infiltrate size** (for every 1 mm increase)	1.60	(1.44–1.79)	< 0.0001	1.19	(1.03–1.36)	0.020
**Perforation status at presentation**						
Not perforated	1		< 0.0001	1		< 0.0001
Impending perforation	11.9	(5.27–26.9)		2.86	(1.11–7.37)	
Perforated and sealed	5.60	(1.44–21.8)		1.57	(0.31–7.76)	
Perforated	41.0	(17.3–97)		9.93	(3.70–26.6)	
**HIV status being positive**	0.85	(0.39–1.85)	0.683			
**Diabetes status being positive**	0.81	(0.34–1.92)	0.630			
**Microbiology**						
No organism detected	1		0.063			
Bacteria	1.48	(0.53–4.14)				
Fungal	2.25	(1.19–4.26)				
Mixed	2.80	(0.86–9.01)				

^a^All crude estimates were adjusted for age and sex. ^b^Final predictive model adjusted for age and sex.

## Discussion

This study describes the clinical history, signs, microbiological etiology, causes, and outcomes of MK in Uganda. Most patients presented with poor vision. At 3 months, 30% had monocular blindness in the affected eye and 1 in 10 lost their eye to infection.

Delayed presentation was common. Very few (7%) presented within 3 days of symptom onset and this had a direct impact on outcomes, as previously reported.^13^ In this study, delayed presentation after adjusting for being a farmer, distance, economic status, education status, trauma, TEM and previous use of other treatment was associated with poor presenting vision. Earlier studies indicate that prompt prophylactic antibiotic can prevent simple corneal abrasions developing into MK, leading to much better outcomes.^17,21,22^ Most late presenters had advanced ulcers, where treatment could do little. We know from prior literature that once an ulcer is advanced, treatment does relatively little to change its course.^23^ From previous studies, it is recommended that treatment of MK should be started as early as possible to achieve optimal outcomes.^17^

Another important cause of poor vision at presentation was Traditional Eye Medicine use. In this study, 60% of the patients reported TEM use. TEM increased the odds of poor presentation by 60% after adjusting for age, sex, being a farmer, economic status, education level, and distance. In our model, some of the effects of TEM seemed to be mediated through delay and organism type. But after adjusting for these, there was still an estimated 40% increase in odds of poor presentation, although the evidence for this association was weak. Many people probably try TEM before attending hospital, as it can be easily obtained within or close to home. In Uganda, TEM is usually made from plant products. This is concerning, as such substances may be toxic or harbor infectious agents, such as fungal spores.^7,24^ Importantly, our patients were open in admitting use of TEM, a widely acceptable practice for treating MK.

Distance was an important cause of poor presenting vision. This included distance to the eye hospital and distance to the nearest HC. This highlighted a major underlying problem of access to health services: the further the HC, the lower the chances of promptly starting appropriate treatment. In our model, even after adjusting for delay, distance was still highly associated with poor presenting vision meaning that there were still other unexplained factors in this relationship.

As reported previously, severity of infection at presentation (vision, perforation status, and infiltrate size) was the strongest predictor of outcome.^23,25,26^ Poor vision at presentation (WHO Snellen categories) was strongly associated with a worse visual outcome. Vision is an easily measurable and reliable prognostic measure that can support lower and mid-level cadres to make the right clinical decisions. A perforated eye at presentation had 10 times greater odds while an eye with an impending perforation had 3 times greater odds of a worse visual outcome compared to a non-perforated eye. This was not surprising because keratoplasty services are currently not available in Uganda. People who presented with threatened or full perforation underwent conjunctival flap or evisceration surgery depending on the extent of the perforation.

Most of our patients presented with large infiltrate and epithelial defect sizes. Such median sizes would be considered severe ulcers in a high-income setting. The epithelial defect size was not included in the analysis because it was highly correlated to the infiltrate size. A large infiltrate size was associated with increased odds of a worse final visual outcome.^25,26^

Most of the affected patients were aged between 31 and 60 years, which are the prime years for economic productivity.^13^ About 70% of the affected people were heads of households and sole breadwinners in their home. Prolonged morbidity due to MK meant that they could not provide for their dependents. In an ongoing study, we have been exploring how MK affects the quality of life and household incomes (unpublished). The prevalence of HIV among our cohort was almost double the national prevalence and diabetes was 4 times the reported prevalence.^27,28^ A high prevalence of HIV has been previously reported in people with MK.^13,29^ HIV and diabetes predispose to MK through immune suppression: we conducted a nested case-control to test for risk factors of MK including HIV and diabetes which have been reported separately.

Understanding the seasonal pattern of presentation is important to prepare a surveillance mechanism and for hospitals to have expectant management. We found that the presentation of MK tended to follow rainfall patterns linked to agricultural activity. This was not surprising since the majority (70%) of patients were farmers. There was little variation in humidity and temperature throughout the year. This region of Uganda has two planting and harvesting seasons, one in each half of the year following rains. Harvesting time is May–July and November–January. These were the periods when we recorded increased numbers of presentations. Farming (especially harvesting) has been linked to ocular trauma which predisposes to MK.^30^ These corresponded with peak presentation to hospital. April has modest farming activity, as people are waiting for the harvesting season and it usually corresponds to Easter holiday. August usually has almost no farming activity since it comes at the end of the harvesting season before the rains come again in September. December had fewer patients presenting, possibly due to the Christmas season.

It remains unclear if this seasonal variation was related to trauma, as there were no clear seasonal differences in the pattern of presentation among patients who reported trauma and those who did not. We were surprised that relatively few patients (29%) reported trauma, although this is consistent with other studies from sub-Saharan Africa (SSA). In an older study from Ghana, 39% of the MK cases reported some form of eye injury prior to onset.^31^ In two separate studies from Tanzania, 24% and 39% of the cases were associated with trauma.^13,32^ These levels are somewhat lower than those from South Asia, where around 75% are associated with an injury.^31,33–36^ The reason for this difference is not apparent.

Ocular microbiology is not performed in many settings in SSA. As part of this study, we undertook to build the capacity of the hospital to provide this service. The overall microbiology yield was 80%. This was a composite of all the microscopy and culture results. Overall culture positive results were 55% similar to the expected yield reported in literature.^16,31^

### Strengths/limitations

This is the first large prospective cohort study in SSA to describe outcomes of MK. Most of the reports have described etiology and presentation.^16,29,31,32^ Only one audit had attempted to describe outcomes.^13^ The large number of patients gave sufficient power to analyze several factors associated with the main outcome measures. It was not possible to follow-up all the patients, with around 20% not having 3-month outcome data; however, no systematic differences were found between those with and without final follow-up data.

## Conclusion

This study provides an understanding of MK epidemiology in Uganda. Majority of patients presented late after having traveled large distances to seek specialist care. Most patients presented with severe ulcers. The outcomes for many were poor, although around half had some improvement of vision with treatment. Predictive factors for these poor outcomes were the state of the eye at presentation. There is need to work on early interventions to prevent patients reaching such a stage where little can be done.

## Supplementary Material

Supplemental Material

## Submission statement

This submission has not been published anywhere previously and that it is not simultaneously being considered for any other publication.
