# Supplementary material for: Epidemiology of Microbial Keratitis in Uganda: A Cohort Study
Source: Ophthalmic Epidemiol. 2019 Dec 12;27(2):121–31. doi: 10.1080/09286586.2019.1700533 (PMC7446037; doi:10.1080/09286586.2019.1700533)
Supplement: Supplemental Material [file IOPE_A_1700533_SM5945.docx]

Supplementary Table 1: Comparison of people lost to follow-up with those with 3 months outcome data

| Variable | Completed 3 months follow-up (n=260) | | | Lost to follow-up (n=53) | | | P value |
| --- | --- | --- | --- | --- | --- | --- | --- |
|  | **Median** | **(IQR)** | **(Total range)** | **Median** | **(IQR)** | **(Total range)** |  |
| Age | 48 | (35-60) | (18-96) | 44 | (34-63) | (18-87) | 0.724 |
|  |  |  |  |  |  |  |  |
| Distance to the Eye hospital in KM | 79 | (50-123) | (0.2-378) | 79 | (58-140) | (1-316) | 0.335 |
|  |  |  |  |  |  |  |  |
| Household population | 7 | (4-8) | (1-28) | 5 | (3-8) | (1-16) | 0.193 |
|  |  |  |  |  |  |  |  |
| Distance to nearest Health Centre in KM | 3 | (1-4) | (0-45) | 3 | (1-4) | (0-35) | 0.909 |
|  |  |  |  |  |  |  |  |
| Presenting vision (Log MAR) | 1.3 | (0.3-2.5) | (0-4) | 1.5 | (0.3-2.5) | (0-4) | 0.273 |
|  |  |  |  |  |  |  |  |
| Infiltrate size (mm)* | 5.19 | (3.28-7.59) | (0.53-13) | 5.59 | (3.32-8) | (0.95-11.96) | 0.610 |
|  |  |  |  |  |  |  |  |
| Epithelial defect size (mm)* | 3.87 | (2.45-6.48) | (0-13.78) | 4.20 | (2.13-7.40) | (0-10.06) | 0.933 |
|  |  |  |  |  |  |  |  |
| Variable | **Count** | **(%)** |  | **Count** | **(%)** |  | **P value** |
| Gender |  |  |  |  |  |  |  |
| Female | 119 | (46%) |  | 20 | (63%) |  | 0.283 |
| Male | 141 | (54%) |  | 33 | (37%) |  |  |
|  |  |  |  |  |  |  |  |
| Occupation |  |  |  |  |  |  |  |
| Farmer | 184 | (71%) |  | 36 | (68%) |  | 0.680 |
| Non-farmer | 76 | (29%) |  | 17 | (32%) |  |  |
|  |  |  |  |  |  |  |  |
| Marital status |  |  |  |  |  |  |  |
| Not marriedƗ | 74 | (29%) |  | 21 | (39%) |  | 0.107 |
| Married | 186 | (71%) |  | 32 | (61%) |  |  |
|  |  |  |  |  |  |  |  |
| Education status |  |  |  |  |  |  |  |
| None | 69 | (26%) |  | 15 | (28%) |  | 0.262 |
| Primary | 130 | (50%) |  | 32 | (60%) |  |  |
| Secondary | 41 | (16%) |  | 4 | (8%) |  |  |
| Tertiary | 20 | (8%) |  | 2 | (4%) |  |  |
|  |  |  |  |  |  |  |  |
| Being head of household |  |  |  |  |  |  |  |
| Yes | 171 | (66%) |  | 41 | (77%) |  | 0.100 |
| No | 89 | (34%) |  | 12 | (23%) |  |  |
|  |  |  |  |  |  |  |  |
| Being HIV positive ǂ |  |  |  |  |  |  |  |
| Yes | 27 | (11%) |  | 10 | (22%) |  | 0.046 |
|  |  |  |  |  |  |  |  |
| Being a Diabetic patient |  |  |  |  |  |  |  |
| Yes | 19 | (8%) |  | 3 | (7%) |  | 0.780 |

* The dimensions exceeded the normal corneal diameter due to some lesions which had eroded into the sclera. Ɨ Not married included single, divorced and widowed. ǂ There seemed to be a systematic difference in HIV proportions among the two groups. Reasons for this are uncertain.


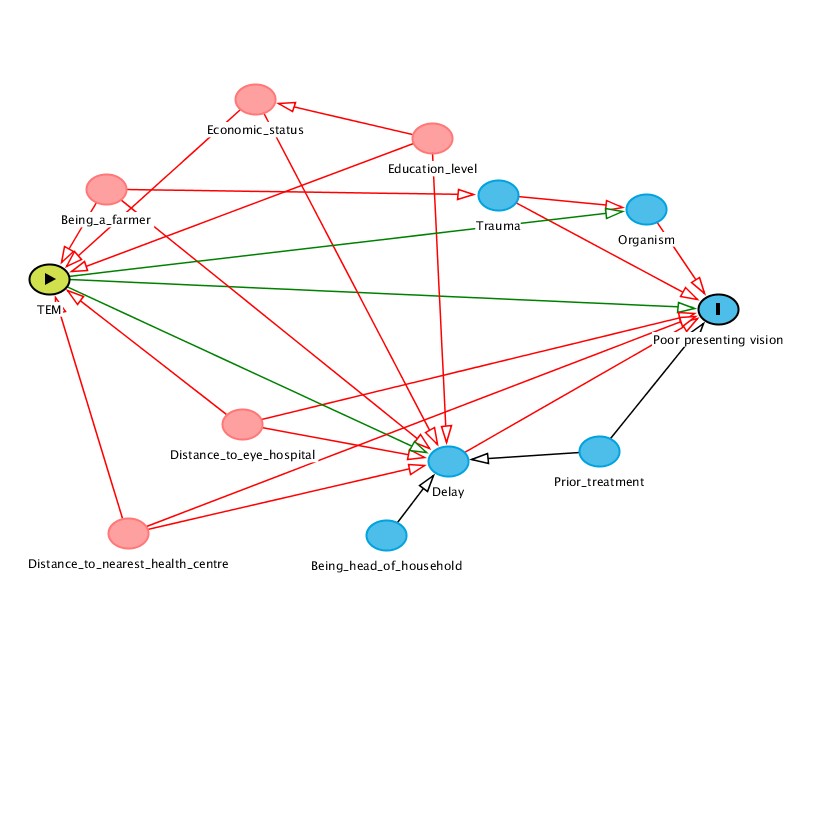
 Supplimentary Figure 1: DAG for a causal effect of TEM on presenting vision

In this DAG, it was necessary to adjust for being a farmer, distance from the eye hospital and distance to the nearest health centre, economic status and education level to be able to correctly estimate the overall effect of using TEM on poor presenting vision. We also separately adjusted for delay and organism type to estimate a direct effect of TEM on poor presenting vision.

**KEY**: In the DAGiity software, Green with a black arrow represents exposure of interest, Green without an arrow represents ancestor of the exposure, Pink is ancestor of the exposure and outcome, Blue is ancestor of the outcome.


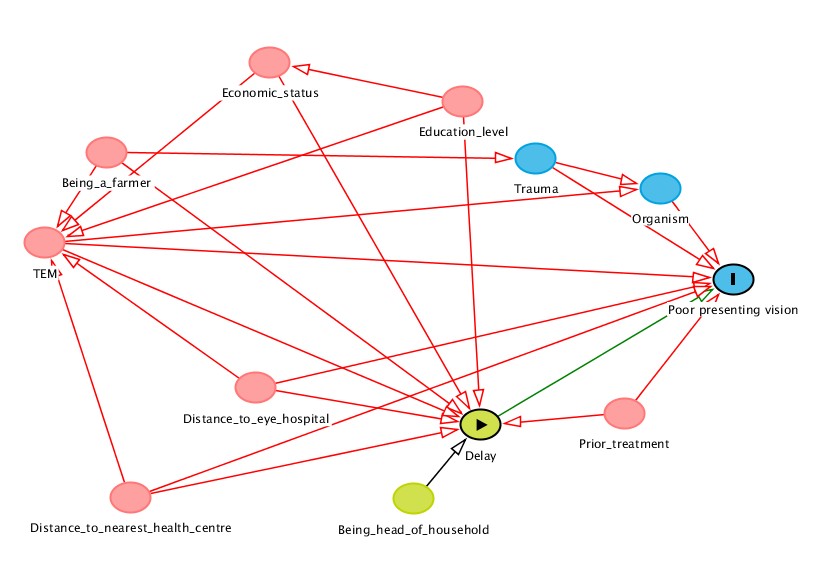


Supplimentary Figure 2: DAG for a causal effect of delayed presentation on presenting vision

In this DAG, it was necessary to adjust for being a farmer, distance, Economic status, Education status, trauma, TEM and previous use of other treatment to estimate the overall causal effect of delayed presentation on presenting vision

**
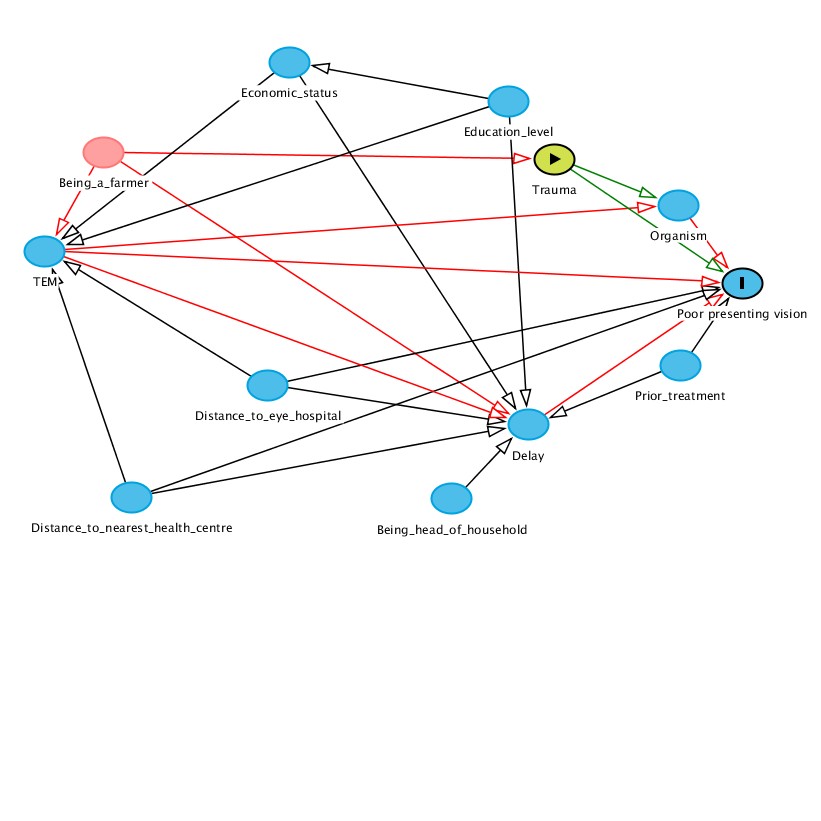
**

Supplimentary Figure 3: DAG for a causal effect of Trauma on presenting vision

In this DAG, it was necessary to adjust for being a farmer, delay, distance, TEM and prior treatment to estimate the overall effect of Trauma on presenting vision


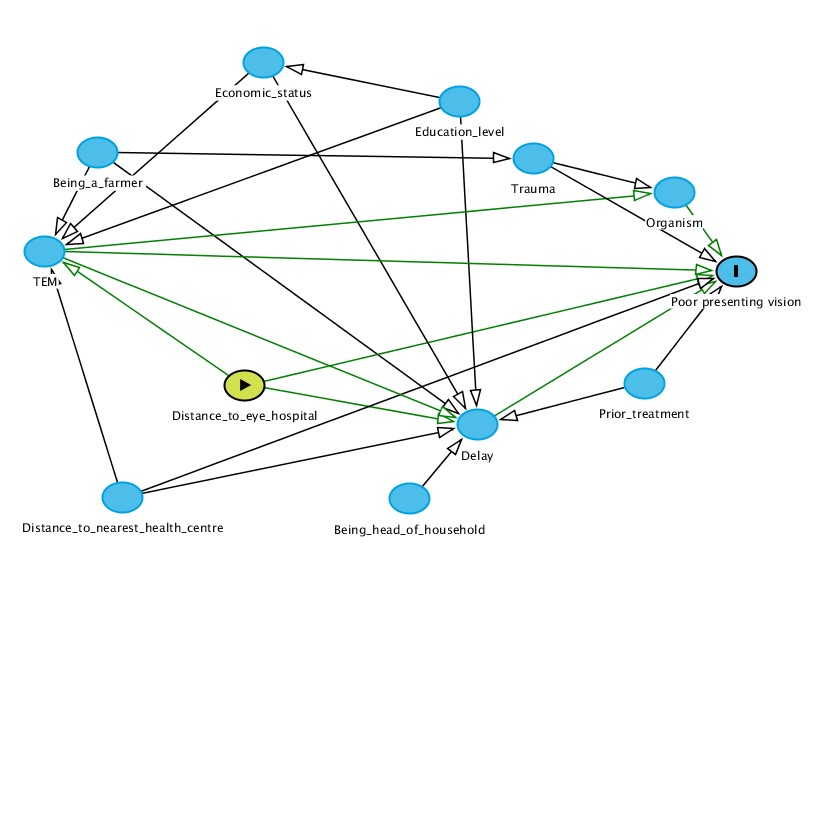


Supplimentary Figure 4: DAG for a causal effect of distance from the eye hospital on presenting vision

In this DAG, it was not necessary to adjust for anything apart from age and sex to estimate the overall causal effect of distance from the eye hospital on presenting vision. However, we separately for delay to estimate a direct effect of distance to the eye hospital on presenting vision.

**
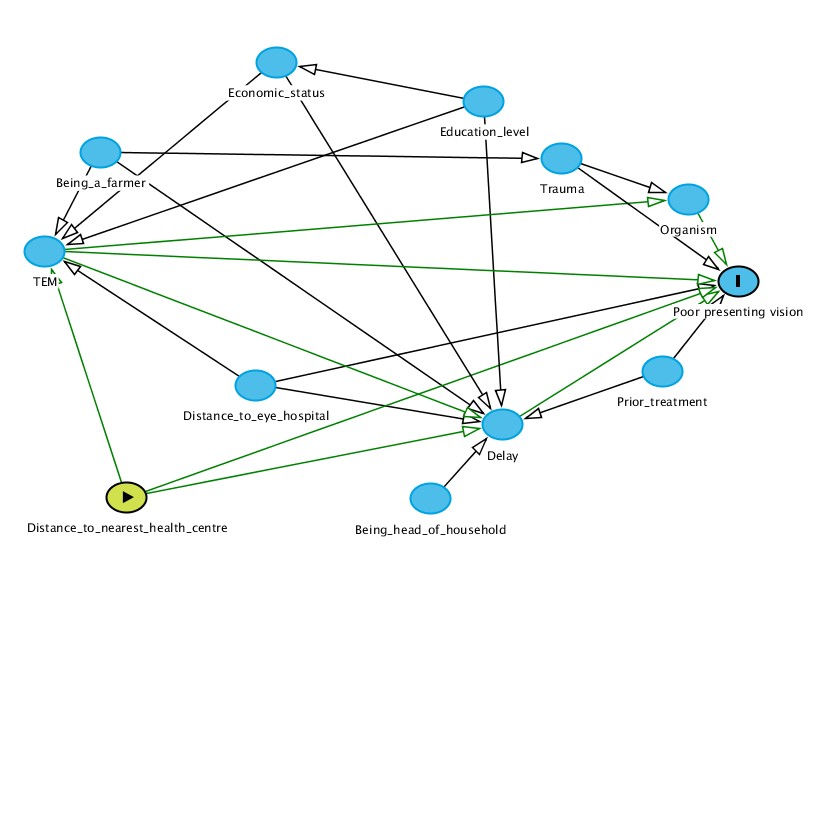
**

Supplimentary Figure 5: DAG for a causal effect of distance from the nearest health centre on presenting vision

In this DAG, it was not necessary to adjust for anything apart from age and sex to estimate the overall causal effect of distance from the nearest health centre on presenting vision. However, we separately adjusted for delay to estimate a direct effect of distance to nearest Health Centre on presenting vision.

**
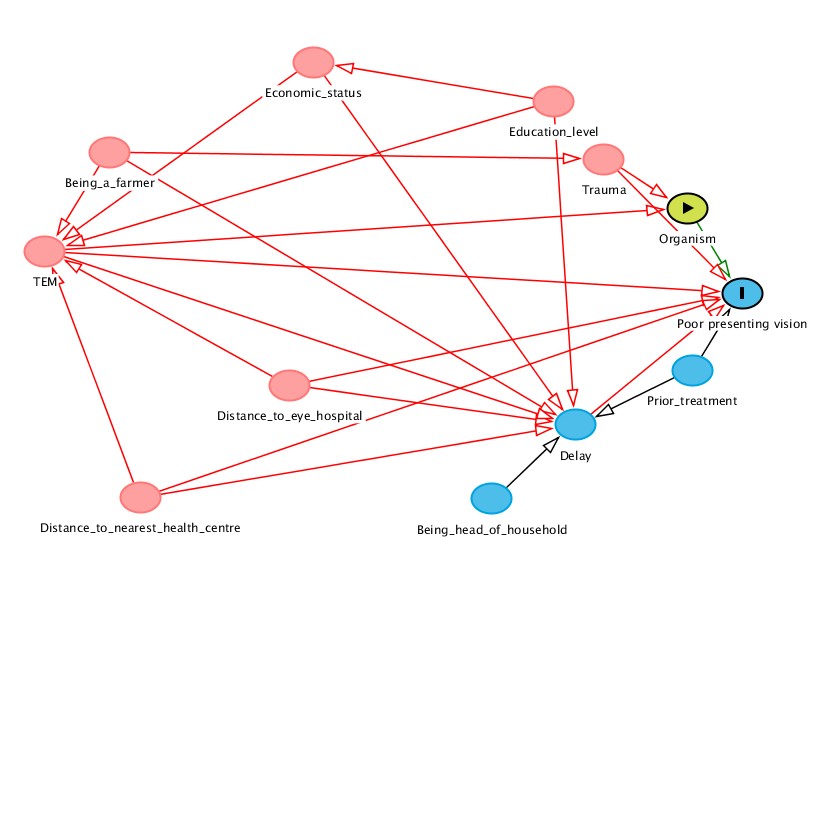
**

Supplimentary Figure 6: DAG for a causal effect of organism type on presenting vision

In this DAG, it was necessary to adjust for Trauma and use of TEM to estimate the overall causal effect of organism type on presenting vision
